# The role of laparoscopy in the propaedeutics of gynecological diagnosis^1^

**DOI:** 10.1590/s0102-865020190010000010

**Published:** 2019-02-14

**Authors:** Gislaine Laperuta Serafim Argentino, Flávia Neves Bueloni-Dias, Nilton José Leite, Gustavo Filipov Peres, Leonardo Vieira Elias, Vitória Cristina Bortolani, Carlos Roberto Padovani, Daniel Spadoto-Dias, Rogério Dias

**Affiliations:** I Assistant Physician, Gynecological Endoscopy and Family Planning Sector, Department of Gynecology and Obstetrics, Botucatu Medical School, Universidade Estadual Paulista (UNESP), Botucatu-SP, Brazil. Conception and design of the study; acquisition, analysis and interpretation of data; technical procedures; manuscript preparation.; II Clinical Assistant Professor, Department of Gynecology and Obstetrics, Botucatu Medical School, UNESP, Botucatu-SP, Brazil. Acquisition, analysis and interpretation of data; technical procedures; manuscript preparation.; III Resident, Department of Gynecology and Obstetrics, Botucatu Medical School, UNESP, Botucatu-SP, Brazil. Acquisition of data,; IV Full Professor, Department of Biostatistics, Botucatu Biosciences Institute, UNESP, Botucatu-SP, Brazil. Statistics analysis.; V Associate Professor III, Department of Gynecology and Obstetrics, Botucatu Medical School, UNESP, Botucatu-SP, Brazil. Conception and design of the study, manuscript preparation.

**Keywords:** Laparoscopy, Endometriosis, Infertility, Female, Pelvic Pain, Techniques, Education, Medical

## Abstract

**Purpose:**

To evaluate agreement between pre- and post-laparoscopy gynecological diagnosis in order to demonstrate the rationality of this minimally invasive technique use in gynecological propaedeutics.

**Methods:**

Retrospective chart review study conducted between March 2010 and October 2016 based on a convenience sample. 315 patients undergoing surgical laparoscopy at the Center of Gynecologic Endoscopy and Family Planning of Botucatu Medical School/UNESP. Pre- and postoperative diagnoses were compared by the diagnosis agreement test considering the proportions of events.

**Results:**

Laparoscopy contributed to diagnosis in 59.6% of infertility cases (P>0.05), in 93.7% of chronic pelvic pain of undetermined origin (P<0.01) and conclusively elucidated the diagnosis of acute abdomen and the ruling out of tubo-ovarian abcess (P<0.05). Laparoscopy also increased the diagnosis of pelvic-abdominal adhesions in 76.7% (P>0.05).

**Conclusion:**

The use of laparoscopy considerably contributed to diagnostic elucidation, especially in cases of undetermined chronic pelvic pain.

## Introduction

 Diagnosis plays a crucial role in clinical practice. It is the basis for developing an adequate treatment plan and establishing effective patient management strategies^1^. Accurate diagnosis reduces the risk of unnecessary therapies and optimizes the use of resources, particularly when they are limited, bringing benefits throughout the medical assistance process^1^ ^,^ ^2^. In the field of Gynecology, a careful diagnosis is especially critical because a great part of the symptoms and diseases that affect women may directly correlate with other specialties. Thus, misdiagnosis can shortly lead to the worsening of a patient’s condition, aggravating morbidity and causing higher costs to the healthcare system^3,^ ^4^.

 Over the past years, laparoscopy has become a powerful propedeutic as well as therapeutic tool of modern gynecological practice. It can reduce the number of innappropiate procedures and unnecessary treatments with very low complication rates^5^ ^-^ ^9^. In conjunction with other propedeutic procedures, laparoscopy may change the diagnostic conclusion in many gynecological cases with increased efficiency in the diagnosis of conditions undetected during previous clinical and ultrasound examinations^5^ ^-^ ^9^ ^,9-^ ^12^.

 Nonetheless, in many countries, medical residency programs in gynecology do not include laparoscopic training, be it for surgical therapeutic or even purely diagnostic procedures. Therefore, this study aimed to assess patients underwent laparoscopy to evaluate the consistency of agreement between pre- and post-laparoscopy gynecological diagnosis in order to demonstrate the rationality of laparoscopy use in gynecological propaedeutics, and thus expand the discussion about the basis of the training of future gynecologists.

## Methods

###  Study design and sample selection 

**Figure 1:**
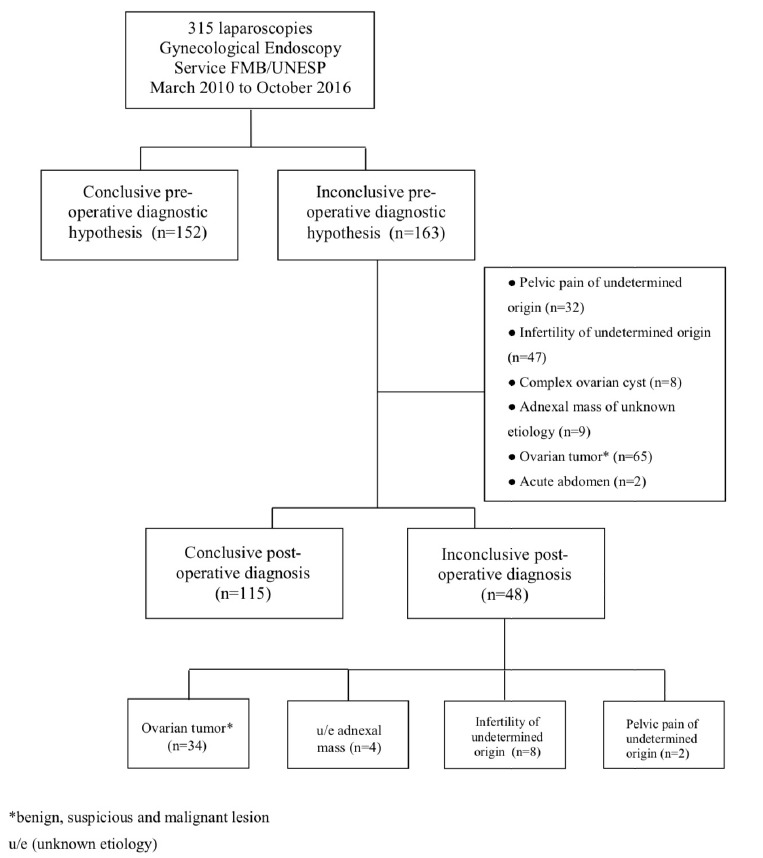
Flowchart of diagnosis in 315 patients who underwent surgical laparoscopy between March 2010 and October 2016 in the Gynecologic Endoscopy Service of Botucatu Medical School/UNESP.

 This retrospective chart review study was conducted to evaluale all patients undergoing surgical laparoscopy at the Center of Gynecologic Endoscopy and Family Planning of Botucatu Medical School/UNESP, Brazil between March 2010 and October 2016 (Fig. 1). Pre- and postoperative data were retrieved from the electronic database of the Botucatu Medical School Hospital/UNESP. The study was approved by the institution’s Committee of Research Ethics and was exempted from the requirement for informed consent as it involved deidentified data acquired during routine care, and did not involve any biological material or contact with the patients.

###  Data collection 

 Data, such as age, body mass index (BMI= kg/m²), parity, abortion, age at menarche, date of last menstruation, menstrual pattern, use of hormone therapy, history of pelvic inflammatory disease (PID), presence of systemic arterial hypertension (SAH), diabetes mellitus, thiroid diseases, dyslipidemia and smoking were transcribed into an Excel spreadsheet for analysis. Information on patients’ clinical complaints, transvaginal-pelvic ultrasound findings, anatomopathological findings, type of surgical procedures performed, and the rate of complications observed before, during and after laparoscopy were also comparatively assessed.

###  Statistical analysis 

 Descriptive statistical analysis was performed. The absolute and relative frequencies of the study parameters were assessed. Mean, standard deviation, and minimum-maximum values of quantitative variables, as well as the absolute frequency and percentage of qualitative variables were estimated. Pre- and postoperative diagnoses were compared by the diagnosis agreement test considering the proportions of events^13^.

## Results

 Patient mean age was 35 years. The majority of women were overweight (BMI= 26.94 ±5.52) and in reproductive age (84.1%) (Table 1). Hormone contraceptives were used by 34.6%, and history of treatment for uterine infection was reported by 11.4% of the patients. The most frequently reported complaint was chronic pelvic pain (34%), followed by dysmenorrhea (15.9%), desire for definitive contraception (15.6%), and desire for reproduction (13%) (Table 2). Eleven per cent of the women complained of significantly increased menstrual flow, while 14.3% had no clinical complaint and were referred to surgery due to incidental image findings, notably regarding adnexal formations. Nineteen per cent of the patients reported more than one clinical complaint of relevance.

**Table 1:** Clinical and epidemiological characteristics of 315 patients who underwent surgical laparoscopy between March 2010 and October 2016 in the Gynecologic Endoscopy Service of Botucatu Medical School/UNESP.

Age	35 (13;72)
BMI	26.94 (±5.52)
Gestation	2 (0;11)
Parity	1 (0;8)
Abortion	0 (0;3)
C-section	0 (0;4)
Menacme	265 (84.1)
Menopause	50 (15.9)
Hormone contraceptive	109 (34.6)
PID history	36 (11.4)
SAH	53 (16.8)
Diabetes	13 (4.1)
Thireoidpathy	18 (5.7)
Dislipidemia	12 (3.8)
Smoking	39 (12.4)

Median (minimum and maximum); Mean values (± standard deviation)

Frequency Distribution in absolute numbers and percentages n(%)

BMI= Body mass index; PID= Pelvic inflammatory disease; SAH= systemic arterial hypertension.

**Table 2:** Clinical characteristics of 315 patients who underwent surgical laparoscopy between March 2010 and October 2016 in the Gynecologic Endoscopy Service of Botucatu Medical School/UNESP

Chronic pelvic pain	107 (34)
Dysmenorrhea	50 (15.9)
Desire for definitive contraception	49 (15.6)
Asymptomatic	45 (14.3)
Reproduction desire	41 (13)
Increased menstrual flow	36 (11.4)
Acute pelvic pain	15 (4.8)
Dyspareunia	14 (4.4)
Irregular menstrual cycle	8 (2.5)
Dyschezia	5 (1.6)
Postmenopausal bleeding	4 (1.3)
Dysuria	1 (0.3)

Frequency distribution in absolute numbers and percentages n(%)

**Figure 2:**
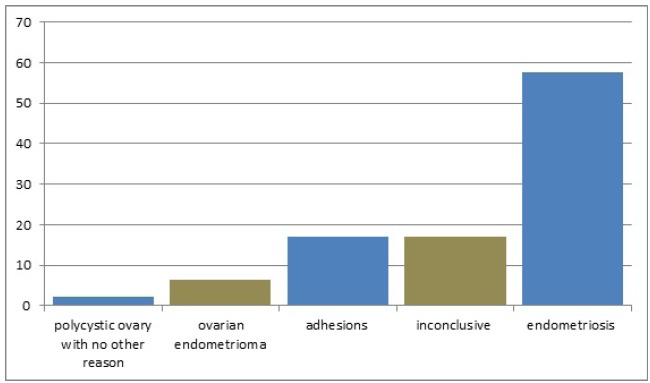
Post-laparoscopy diagnosis of preoperative undefined infertility.

**Figure 3:**
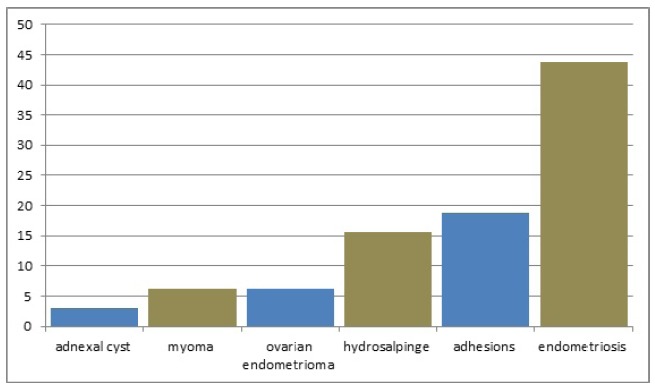
Post- laparoscopy diagnosis of preoperative chronic pelvic pain of undetermined origin.

 Laparoscopy was inconclusive in 17% of the patients assessed due to infertility with no apparent cause. However, in 83% of these cases it was essential for establishing the diagnosis of polycystic ovary (2.1%) with no other reason for infertility, ovarian endometrioma (6.4%), adhesions (17%), and endometriosis (57.5%) (P>0.05) (Fig. 2). The diagnosis of chronic pelvic pain of undetermined origin was postoperatively diagnosed as adnexal cyst (3.1%), myoma (6.3%), ovarian endometrioma (6.3%), hydrosalpinge (15.6%), adhesions (18.7%), and endometriosis (43.7%) (Fig. 3).

 The pre-laparoscopy diagnosis of complex ovarian cyst was related with the post-laparoscopy diagnosis of hemorrhagic corpus luteum (37.5%), ovarian tumor (ovarian teratoma on anatomopathology) (50%), and retention cyst (12.5%). In 40% of the cases with a preoperative diagnosis of ovarian tumor, and in 77.8% of cases with a diagnosis of adnexal mass of unknown etiology, laparoscopy detected uterine myoma, endometriosis, adhesions and tubo-ovarian abcesses, but with no statistical differences (Table 3).

**Table 3:** Distribution of agreement between pre- and postoperative diagnoses.

PRE-diagnosis	POST-diagnosis		Total	P value
	Agreement	Disagreement		
Infertility	40.4%	59.6%	47	>0.05
Pelvic pain	6.3%	93.7%	32	**<0.01**
Complex ovarian cyst	12.5%	87.5%	8	**< 0.05**
Ovarian tumor	60.0%	40.0%	65	>0.05
u/e Adnexal mass	22.2%	77.8%	9	>0.05
Acute abdomen	0.0%	100.0%	2	**<0.05**
Tubo-ovarian abcess	0.0%	100.0%	2	**<0.05**
Adhesions	23.3%	76.7%	7	>0.05

Diagnosis agreement test (pre-post) considering the proportions of events

P<0.05 statistical significance

u/e (unknown etiology)

 In general, laparoscopy contributed to diagnosis elucidation in 59.6% of infertility cases (P>0.05), 93.7% of chronic pelvic pain of undetermined origin (P<0.01), 87.5% of complex ovarian cyst cases (P<0.05), 40% of ovarian tumor cases (P>0.05), and 77.8% of adnexal mass of unknown etiology cases (p>0.05) (Table 3). Furthermore, in this study, laparoscopy changed the preoperative diagnosis of acute abdomen to tubo-ovarian abcess (P<0.05), and ruled out the preoperative diagnostic hypothesis of tubo-ovarian abcess by revealing an ectopic pregnancy and apendicitis (P<0.05). Laparoscopy also determined a 76.7% increase in the diagnosis of pelvic-abdominal adhesions (P>0.05). The rate of complications in our study was low, with emphasis on bladder (0.6%), ureter (0.3%) and intestinal injuries (0.3%).

## Discussion

 Compared to conventional open surgery, laparoscopy has numerous advantages, such as (1) less postoperative pain, (2) shorter hospital stay, (3) lower rates of postoperative complications, (4) early return to daily activities, and (5) better esthetic effect. These benefits combined decrease the direct and indirect costs related to the surgical procedure^3^ ^,^ ^5^ ^,^ ^7^ ^,^ ^11^. In cases where the etiology of the condition is not fully clear, laparoscopy has been shown to be a safe and reliable adjunct to gynecological diagnosis that may spare patients an exploratory laparotomy and its greater risks of complications^14^ ^-^ ^19^.

 To some authors, laparoscopy should be an integral part of the evaluation of women with pelvic pain of undetermined origin due to its safety and reliability^20^. Others additionally state that laparoscopy allows for a much more accurate gynecological diagnosis as it may confirm or rule out clinical impressions, establish a diagnosis, follow and explore the course of the disease as well as change or complement treatment^21^ ^,^ ^22^. Several comparative studies have shown that the use of laparoscopy increased the rate of success in the diagnosis of different gynecological conditions^9^ ^,^ ^22^ ^,^ ^23^. Despite the evidence reported in the literature, in many countries, medical residency programs aiming at providing basic introductory training in diagnostic laparoscopy do not integrate as part of the curriculum. In countries were those programs were implemented there is no standardized training adopted leading to a very heterogeneous formation of future professionals^24^ ^-^ ^26^. 

 Regulatory bodies have already emphasized the need of expanding validated training programs to prepare professionals for the increasing use of laparoscopy before progression to real procedures^25^. Nevertheless, difficulties in implementing a training program for residents in gynecology are encountered in many countries. The main obstacles reported are lack of planning and structure within institutions, cost constraints, shortage of skilled professionals available for teaching and guide residents and limited residents’ working hours^24^ ^,^ ^25^ ^,^ ^27^ ^-^ ^30^.

 It is estimated that 73% of programs lead off laparoscopic skills in North America but only 29% of residencies provide a structured surgical curriculum and only 55% of residency programs have facilities for training in laparoscopy in the United States^26^ ^,^ ^31^ ^,^ ^32^. Moreover, a recent study on accredited North American Obstetrics and Gynecology residency programs revealed that more than 40% were dissatisfied with their current laparoscopy training^33^. As a matter of fact, despite residency programs are trying to incorporate simulation into the resident training curriculum to supplement the hands-on experience gained in the operating room, this simulation laboratories continue to be under utilized by surgical trainees^29^. In most countries, including Latin America, there is not even teaching models for laparoscopic skills or validated tools for its evaluation during residency^32^.

 In this study, after excluding the cases of adnexal and/or ovarian diseases (adnexal masses of unknown etiology, ovarian tumors and complex ovarian cysts) which require histopathological confirmation and cannot be accurately identified surgically, diagnostic elucidation after laparoscopy occurred in 93.7% of the cases of pelvic pain of unknown etiology (P<0.01), and 59.6% of the cases of infertility with no apparent cause (p>0.05). Furthermore, laparoscopy was conclusive in the diagnosis of acute abdomen, even though there was a low number of cases with this condition in our series (P<0.05).

 The introduction of laparoscopy into clinical practice has opened up new avenues for the diagnosis and management of chronic pelvic pain. It is estimated that more than half of patients with a normal preoperative pelvic examination will present abnormal findings during the laparoscopic procedure^11^ ^,^ ^34^. The literature shows that in women with chronic pelvic pain undergoing laparoscopy, the diagnosis may remain inconclusive in approximately 35% of cases, and endometriosis and adhesions can be diagnosed in 33% and 24%, respectively^10^ ^,^ ^11^ ^,^ ^34^ ^,^ ^35^. These findings represent about 90% of all laparoscopies in women with pelvic pain suggesting that the predominant role of laparoscopy in the evaluation of these patients is to diagnose or rule out endometriosis and adhesions.

 Except in cases of endometrioma, ovarian retention syndrome and ovarian residual syndrome, ovarian cysts are not a common cause of chronic pelvic pain. The laparoscopic assessment of patients with chronic pelvic pain reveals ovarian cysts in only 4% of all cases excluding endometriomas^11^. Endometriosis, in turn, is a common laparoscopic diagnosis in patients with chronic pelvic pain, found in 15% to 80% of women undergoing surgery for chronic pelvic pain^12^ ^,^ ^36^. 

 Similarly, endometriosis is estimated to affect up to 50% of infertile women, and its severity appears to correlate with reduced fertility^37^. Infertility is the classical indication for propedeutic/therapeutic laparoscopy, which is indispensable to elucidate cases with no apparent cause^38^ ^-^ ^40^. According to non-controlled retrospective studies, diagnostic laparoscopy performed after several failed ovulation induction treatment cycles reveal significant pelvic pathology amenable to surgical treatment^40^. Laparoscopy indicates intra-abdominal abnormalities in 36%-68% of cases, even after normal hysterosalpingography^38^ ^,^ ^39^. Depending on the severity of laparoscopic findings, the initial treatment decision may be replaced by direct laparoscopic intervention, a laparotomic approach to fertility restoration or in vitro fertilization. This implies that, in addition to being a clinically important diagnostic tool, laparoscopy is essential for infertility treatment decision making^39^. 

 In this study, laparoscopy also proved to aid the diagnosis of tubo-ovarian abcess (P<0.05). Despite the small number of cases, these findings corroborate the role of laparoscopy as a specific clinical criterion for the diagnosis of complicated pelvic inflammatory disease^15^ ^,^ ^41^. Although no statistical difference was reached, laparoscopy increased in 76.7% the diagnosis of pelvic-abdominal adhesions, demonstrating that preoperative propedeutics is still ineffective to establish the diagnosis of this condition. Adhesions are commmon etiologic factors for infertility, dyspareunia, intestinal obstruction and chronic pelvic pain albeit their role in the physiopathology of pain remains unclear^42^. Laparoscopy in 1,061 patients revealed that pelvic adhesions (found in 32.5% of cases) is the most common cause of chronic pain^34^ ^,^ ^43^. 

 The use of laparoscopy can reveal treatable conditions, not detected using other methods, with a very low rate of complications. In our study, the rate of potentially severe complications ranged from 0.3% to 0.6%. A survey of 6.451 laparoscopic procedures showed an overall complication rate of 0.65% (42/6451). However, this rate rose to 0.80% (39/4865) when surgical laparoscopy was compared to merely diagnostic laparoscopy that was associated with a complication rate of 0.19% (3/1586) (P<0.001)^44^.

 The benefits of this minimally invasive technique indicate that an in-depth discussion on reshaping medical residency programs is necessary as to adjust them to the new technology available as well as to today’s reality. Given its propedeutic nature and association with very low complications risks, diagnostic laparoscopy should be routinely addressed in the training of future gynecologists. All efforts should be made so that health policies contemplate the dissemination and increasing use of laparoscopy, which has been demonstrated to offer numerous advantages throughout the medical assistance system, especially in the field of gynecology.

###  Take home messages

- Since it is not included in the training of resident doctors in most services worldwide, laparoscopy’s potential for development in still considerable;
- The propedeutic role of laparoscopy for elucidating the diagnosis of several conditions of undetermined origin, notably complaints related to infertility and chronic pelvic pain, should not be disregarded;
- Programs of medical residency in gynecology including training in the use of propedeutic/diagnostic laparoscopy should be encouraged by medical care improvement policies. This can bring direct and indirect benefits, besides reducing costs throughout the healthcare system;
- Training in therapeutic laparoscopy should be provided in specialized centers because the learning curve, despite being reproducible, takes quite long and depends on the number of procedures performed by the surgeon.
